# Electronic communication during nonwork time and withdrawal behavior: An analysis of employee cognition-emotion-behavior framework from Chinese cultural context

**DOI:** 10.3389/fpsyg.2022.1010197

**Published:** 2022-09-27

**Authors:** Ganli Liao, Miaomiao Li, Jielin Yin, Qianqiu Wang

**Affiliations:** School of Economics and Management, Beijing Information Science and Technology University, Beijing, China

**Keywords:** Chinese cultural context, electronic communication during nonwork time, employee withdrawal behavior, cognition, regulatory emotional self-efficacy

## Abstract

Although a large number of literatures have explored the relationship between electronic communication during nonwork time and individual perception and behavior under the Western culture background, we still have some limitations on this topic under the cultural background of collectivism, dedication and “Guanxi” in China. Different from Western organizations, Chinese employees tend to put work first and are more inclusive of handling work tasks during nonwork time. This type of communication during nonwork time can significantly affect employees’ cognition, emotion and behavior. From the perspective of Job Demands-Resources theory, this study constructs a double-edged (U-shaped) model between electronic communication during nonwork time and employee withdrawal behavior. Sample data were collected from 516 employees with clear working time boundaries in China. The results indicate that electronic communication during nonwork time has a U-shaped effect on employee withdrawal behavior and an inverted U-shaped effect on employee’s cognition, namely job engagement. Job engagement plays a mediating role between them. Moreover, regulatory emotional self-efficacy has a moderating effect on the relationship between electronic communication during nonwork time and job engagement. These findings not only provide theoretical and practical implications for managers and employees on how to reduce withdrawal behaviors, but also advance our understanding of electronic communication during nonwork time in Chinese cultural context.

## Introduction

With the popularization of mobile communication technology and mobile internet, it is increasingly convenient for people to use devices for telecommunication, and electronic communication has become an indispensable component of people’s lives (Schlachter et al., 2018). Different from Western culture, the values of collectivism, dedication and “Guanxi” in the Chinese context led most employees to put the interests of the organization in the first place (Li and Nesbit, 2014). They are more willing to sacrifice personal time to achieve organizational goals when their leaders assign heavy job tasks. As a result, an increasing number of Chinese employees are dealing with work-related matters during nonwork time and even keeping their mobile phones on 24 h a day. Especially in the post-COVID-19 era, “online” work time has gradually penetrated into nonwork time, making the boundaries between the work domain and the family domain increasingly indistinct in China. New work modes such as the “996” working system, “WeChat,” and “Tencent Meeting” transcend the limitations of time and space and lower the communication costs within organizations. At this time, employees using remote intelligent terminals for communication for work during nonwork time has become a universal phenomenon in organizations (Boswell and Olson-Buchanan, 2007), which is called electronic communication during nonwork time (Butts et al., 2015).

Studies about electronic communication during nonwork time have become a new topic in the field of organizational behavior, and mainly focus on exploring the impact on individual’s emotion, cognition and behavior (Richardson and Thompson, 2012; Becker et al., 2018). However, the conclusions of these studies are inconsistent. Most researches proposed that electronic communication during nonwork time has negative effects on employees’ emotion, cognition and behavior, such as negative emotional responses, work–family conflict and poor sleep quality (Duxbury et al., 1992; Derks et al., 2014; Lanaj et al., 2014; Butts et al., 2015; Ferguson et al., 2016). While other scholars claimed that electronic communication during nonwork time has positive effects, such as work autonomy, work efficiency, job satisfaction and interpersonal interaction (Chesley, 2010; Diaz et al., 2012; Fonner and Roloff, 2012; Fujimoto et al., 2016). Therefore, some scholars proposed that electronic communication during nonwork time may have both harms and benefits to employees, rather than a simple linear impact. For example, Ma and Ma (2021) proposed that there was an inverted U-shaped relationship between electronic communication during nonwork time and work-family gains. Reinke and Ohly (2021) believed that electronic communication during nonwork time involved negative and positive effects on employee wellbeing. However, further studies are needed to investigate the nonlinear relationship between electronic communication during nonwork time and negative behaviors of employees. Employee withdrawal behavior refers to a series of negative behaviors that employee keeps away from the organization and weakens the connection with the organization when he/she perceives an uncomfortable situation in the organization (Gupta and Jenkins, 1991). In recent years, some scholars have started to pay attention to the influence of work-related variables on employee withdrawal behavior (Schabram and Maitlis, 2017). At this time, will employee withdrawal behavior be influenced by electronic communication during nonwork time?

To answer this question, this study aims to explore the relationship based on the Job Demands-Resources (JD-R) theory (Demerouti et al., 2001). The JD-R theory points out that the characteristics of any job can be divided into job resources and job demands. Job resources refer to all physical, psychological, social and organizational resources that can reduce the physical and psychological costs, accelerate the realization of job objectives, and promote individual growth and development. Job demands refer to all physical, psychological, social or organizational demands that require continuous physical and psychological effort or cost, such as work overload and role conflict. Job resources and job demands can stimulate two potentially different mental processes, namely, gains or losses (Schreurs et al., 2014). Hence, the two processes of gains and losses can provide theoretical explanations for the double-edged relationship between electronic communication during nonwork time and withdrawal behavior (Chen et al., 2017). On the one hand, electronic communication during nonwork time is “technology assisting in the remaining work,” and it can be regarded as job resource, which allows employees to meet their social needs, improves personal competitiveness, and increases production efficiency (Zhang et al., 2019). Previous studies indicated that these job resources can promote employees’ job engagement and make employees more willing to invest more time and energy in their work (Syrek et al., 2018; Shin et al., 2020), thus reducing employee withdrawal behavior. On the other hand, continuous “work online” state also further blurs the boundaries between work and family and such infringement and conflict cause role stress, which decreases employee’s job engagement (Moura et al., 2014). Therefore, this study attempts to further explore whether there is an inverted U-shaped relationship between electronic communication during nonwork time and job engagement, what are the boundary conditions of this relationship, and whether job engagement can further affect employee withdrawal behavior. According to the JD-R theory, the job resources and job demands of employees can produce challenge stress and hindrance stress, respectively, (Broeck et al., 2010; Bakker and Demerouti, 2017). However, no matter what kind of stressor, individuals are prone to emotional exhaustion, anxiety, fear and other negative emotions (Dawson et al., 2016; Schneider et al., 2017). Subsequently, scholars began to pay attention to regulatory emotional self-efficacy, which refers to the ability of individuals to regulate their emotions, including positive emotion regulation and negative emotion regulation (Bandura et al., 2003). The empirical evidence suggests that the regulatory emotional self-efficacy is crucial to individual attitudes, cognitions and behaviors (Caprara et al., 2008; Sui et al., 2021). For example, under the same level of job satisfaction, employees with high level of regulatory emotional self-efficacy have higher level of job engagement (Lan et al., 2020). Hence, we argue that regulatory emotional self-efficacy may moderate the relationship between electronic communication during nonwork time and job engagement.

To sum up, we attempt to make three major contributions in this study. First, based on the JD-R theory, this study investigates the relationship between electronic communication during nonwork time and employee withdrawal behavior in Chinese cultural context, such as collectivism, dedication and “Guanxi,” greatly enriching the relevant topics in the above fields. Second, different from the previous linear relationship, this study explores the U-shaped relationship between electronic communication during nonwork time and employee withdrawal behavior, which enriches and improves the research on them. Third, the study uses job engagement as mediating variable and regulatory emotional self-efficacy as moderating variable, which help people better understand the influence mechanism between electronic communication during nonwork time and employee withdrawal behavior.

## Theory and hypotheses

### Electronic communication during nonwork time and employee withdrawal behavior

Currently, scholars generally define electronic communication during nonwork time as the use of electronic tools by employees to handle work-related materials or maintain contact with people during nonwork time (Richardson and Thompson, 2012; Butts et al., 2015; Becker et al., 2018). As for the definition of communication tools, some scholars define them as computers, iPads or telephones (Derks et al., 2016; Ferguson et al., 2016), while most studies still define communication tools as a broader range of channels, namely electronic communication technologies (Boswell and Olson-Buchanan, 2007), including practical tools such as emails, “WeChat,” “Tencent Meeting” and other Apps. Studies on electronic communication during nonwork time were mainly based on the Work Family Boundary, Ego Depletion, and Conservation of Resources theories (Becker et al., 2018; Von-Bergen and Bressler, 2019; Zhang et al., 2019;), and they provided empirical evidence for the negative or positive impacts on individuals (Chesley, 2010; Derks et al., 2014; Butts et al., 2015; Ferguson et al., 2016; Fujimoto et al., 2016). However, some scholars believe that the relationship between electronic communication during nonwork time and employees’ emotion, cognition and behavior is not just a simple linear relationship, but should be more complex, showing a double-edged effect (Ma and Ma, 2021; Reinke and Ohly, 2021).

Employee withdrawal behavior is the extension and development of withdrawal behavior in the area of organizational management. It refers to a series of covert and retaliatory passive behaviors done intentionally by employees to avoid job task, keep away from the organization, and weaken their connection with colleagues when they perceive a repulsive situation in the organization (Sliter et al., 2012; Khan et al., 2022). Employee withdrawal behavior starts with occasional daydreams, gradually evolves into lateness, absence, and turnover intention (Lehman and Simpson, 1992; Shaukat and Khurshid, 2021). Employee withdrawal behavior has negative impacts on the development of individual and organization, such as knowledge sharing willingness, job performance, organizational change and so on (Sheridan, 1985; Fugate et al., 2012; Tian et al., 2021). Therefore, scholars began to explore what strategies organization and individual should adopt to effectively reduce employee withdrawal behavior (Cole et al., 2010; Tian et al., 2021).

Based on the JD-R theory, job characteristics of any job can be divided into job resources and job demands. These two aspects further stimulate two potentially different mental processes that affect employees in diametrically opposite ways, namely, bringing gains or losses (Demerouti et al., 2001). The path of gain means the job resources can lead to positive outputs, which can facilitate job engagement, reduce interpersonal indifference, and create high performance (Salmela-Aro and Upadyaya, 2018). The path of loss refers to the situation where long-term excessive job demands continuously consume employees’ physical strength and energy and employees cannot obtain effective resource support, which could lead to health problems or job burnout (Bakker and de Vries, 2021). This study proposes that electronic communication during nonwork time has a U-shaped effect on employee withdrawal behavior. In fact, moderate electronic communication during nonwork time can increase individual interaction and communication. Under the traditional Chinese culture, it can promote the “Guanxi” (relationship) between employees and other members, which can be regarded as job resources (Wu et al., 2019). This type of job resources can assist individuals in completing job tasks, that is, it can increase the flexibility and sense of control over work, transcend the limitations of work in time and space, and enable individuals to complete their work more flexibly and independently (Mazmanian et al., 2013). This efficient pattern of work, electronic communication during nonwork time increases the frequency of communication between employees and work-related parties, strengthens the connection between employees and other people, impels employees to establish a sound social network (Ren and Chadee, 2017; Lv et al., 2022). Therefore, all these are helpful to motivate employees’ work passion, improve the perceived organizational support and organization commitment, and promote organizational citizenship behaviors, which can further reduce the behaviors such as lateness, absenteeism and turnover, namely, the employee withdrawal behavior (Knippenberg et al., 2007; Eder and Eisenberger, 2008; Somers, 2009; Smith et al., 2016).

However, as the frequency of electronic communication during nonwork time keeps increasing, excessive electronic communication seriously prolongs the work time of employees and weakens the psychological detachment of employees because they are unable to keep a distance from work during nonwork time (Park et al., 2011). In the context of Chinese culture, employee will put the collective interests first and spend more leisure time to deal with the job task. Consequently, the boundary between work and family is gradually blurred, and the resource allocation mechanism for work and family is destroyed, making it hard for employees to recover resources in a timely manner and making them fall into the spiral effect of resource loss (Gadeyne et al., 2018). Information overload, extra workload, and time and energy spent in non-working time make employees fall into job burnout, resulting in individual anxiety, lassitude and other negative mental states (Banghart et al., 2018; Reinke and Ohly, 2021). Previous studies have shown that job burnout can increase employee’s turnover intention, also known as job withdrawal behavior (Shaukat and Khurshid, 2021). Moreover, the job demands that goes with frequent electronic communication during nonwork time will cause employee’s work stress (Ter-Hoeven et al., 2016), and consume their positive emotions and other physical and mental resources, which will lead to emotional exhaustion (Derks et al., 2014). Specifically, a study by Chong et al. (2020) showed that emotional exhaustion was one of the important factors leading to employee withdrawal behavior. Accordingly, employees are more likely to release stress and relieve fatigue through lazing, slacking and other withdrawal behaviors (Boswell and Olson-Buchanan, 2007). Hence, high electronic communication during nonwork time has a positive effect on employee withdrawal behavior. Thus, we propose the following hypothesis:

*Hypothesis 1*: There is a U-shaped relationship between electronic communication during nonwork time and employee withdrawal behavior.

### Mediating effect of job engagement

Schaufeli et al. (2002) proposed that job engagement is a relatively stable positive state where individuals are willing to be devoted to work and produce high performance based on their perceptions of belonging and identification to the organization. It is characterized by vitality, dedication and focus. Vitality means that the individuals have abundant energy and physical strength at work. Dedication means that individuals devote themselves to their work with enthusiasm. Focus means that individuals are not easily disturbed by the external environment and are highly engaged in work. Previous studies revealed that job engagement can be considered as the degree of integration between employees and their job roles and their performance will be better if they immerse themselves more into their job roles (Rothmann and Jordaan, 2006; Wang et al., 2019).

This study indicates that job engagement mediates the U-shaped relationship between electronic communication during nonwork time and employee withdrawal behavior. As mentioned above, electronic communication during nonwork time has two attributes, job resources and job demands, which may lead to two different results in the impact on job engagement. On the one hand, when the duration of electronic communication during nonwork time is short and the frequency is low, moderate communication for work can improve the permeability and flexibility of boundaries between work and family domains, and make the flow resources between different domains more frequent (Duxbury et al., 2014). As a result, the individual’s ability to control the environment is enhanced (Haynes, 2008). It can be regarded as a job resource that keeps employees energized, dedicated, and focused in the organization. And it also helps employees obtain a sense of control over work progress, increases their job engagement and makes them more willing to focus on work and produce high performance (Owens et al., 2016). The empirical evidences show that job engagement could reduce employee withdrawal behavior (Aggarwal et al., 2020; Garg and Singh, 2020), which indicate that job engagement had a mediating effect between moderate electronic communication during nonwork time and withdrawal behavior. On the other hand, when the level of electronic communication during nonwork time is high, employees need to spend a lot of personal time to deal with job tasks. This extra workload lengthens the working hours and increases the work stress, which leads to the continuous consumption of employees’ positive emotions and behaviors (Derks et al., 2014; Schneider et al., 2017). As a result, the negative emotions of employees keep increasing, and they will gradually lose their enthusiasm and reduce the motivation in the future work (Bandura et al., 2003; Fonner and Roloff, 2012), which will lead to the decrease of job engagement (Shkoler and Kimura, 2020). According to Shusha (2013) research, employees with lower job engagement have higher turnover intention, absence and avoidance behaviors (i.e., job withdrawal behavior). Thus, the following hypotheses can be stated:

*Hypothesis 2*: There is an inverted U-shaped relationship between electronic communication during nonwork time and job engagement.

*Hypothesis 3*: Job engagement mediates the U-shaped relationship between electronic communication during nonwork time and employee withdrawal behavior.

### Moderating effect of regulatory emotional self-efficacy

The research on regulatory emotional self-efficacy dates back to 1990s, Caprara et al. (1999) found that there are great differences between individuals in the management of daily emotional experience, because of individual differences in management skills as well as the capability to regulate emotions. Moreover, Bandura et al. (2003) also emphasized the individual’s ability to manage emotional states when defining the regulatory emotional self-efficacy. They proposed that the regulatory emotional self-efficacy mainly includes the ability to recognize emotional states, the ability to understand the feelings of others, and the ability to manage the expression of positive and negative emotions. It refers to a degree of confidence in whether individuals can effectively regulate their emotional state.

Consistent with the definition of regulatory emotional self-efficacy, this study examines whether the strength of the inverted U-shaped relationship between electronic communication during nonwork time and job engagement differs with high and low levels of regulatory emotional self-efficacy. Studies have shown that regulatory emotional self-efficacy can affect individual behavior by influencing their cognition, motivation and emotion, and play an important role in regulating individual personality and behavior (Alessandri et al., 2015; Mesurado et al., 2018). For example, the research of Bandura et al. (2003) showed that individuals with high regulatory emotional self-efficacy could effectively cope with pressure, improve interpersonal relationship quality and increase subjective wellbeing (Jin et al., 2020). When employees experience moderate non-work time electronic communication, individuals with higher regulatory emotional self-efficacy can ease emotional tension and maintain self-regulation mechanism (Zhang et al., 2019). They can effectively perceive and actively make use of the job resources provided by electronic communication during nonwork time, reduce the work stress, and promote the generation of positive work emotions. These job resources and positive emotions help individuals better realize their self-worth, thus enhancing their job engagement (Lan et al., 2020). However, when individuals experience high level of electronic communication during nonwork time, high level of regulatory emotional self-efficacy of employees are less likely to engage in their jobs. We argue that it may be because employees with high regulatory emotional self-efficacy are able to effectively cope with the work stress and job burnout (Alessandri et al., 2018; He et al., 2021). They take advantage of the positive events and emotions in the family areas to balance the personal cognition and behavior, so as to self-digest the difficulties caused by electronic communication during nonwork time. At this time, they are more likely to shift their focus from work to life, thus reducing the job engagement. Thus, we propose the following hypothesis:

*Hypothesis 3*: Regulatory emotional self-efficacy moderates the inverted U-shaped relationship between electronic communication during nonwork time and job engagement. That is, the inverted U-shaped relationship is stronger with high regulatory emotional self-efficacy than those with low regulatory emotional self-efficacy.

**Figure 1:**
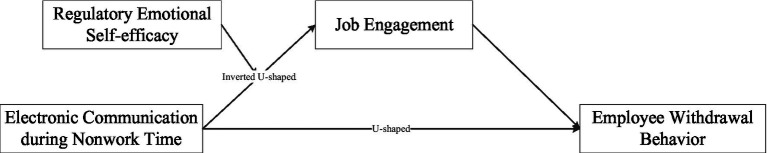
Theoretical model.

The theoretical model is shown in Figure 1.

## Materials and methods

### Samples and collection

Based on the Chinese cultural background of collectivism, dedication and “Guanxi,” the research sample were collected from state-owned enterprises and public institutions with obvious Chinese cultural characteristics. In this study, the simple random sampling was adopted, and the questionnaire was distributed to the target group in the form of email, telephone, web link, APPs and so on. These methods ensured the randomness and universality of the sample. The process of data collection lasted about 4 months (2021.2–2021.6). The respondents were mainly from 6 state-owned enterprises and public institutions in Beijing, Hebei, Guangdong and Jiangsu provinces in China, involved intelligent manufacturing, new energy, financial service and other industries. A total of 723 questionnaires were collected. In the screening process of samples, participants from foreign companies and private enterprises were excluded. Moreover, since electronic communication during nonwork time requires employees to have clear working time boundaries, we have created another selection criterion specifically titled “Do you have clear working time boundaries? (e.g., from 9 a.m. to 5 p.m.).” Based on these selection criteria, 86 questionnaires without clear working time boundaries were removed. Meanwhile, in order to ensure the accuracy of the results, 121 questionnaires with a deletion rate of more than 10% were also excluded. Finally, we collected 516 valid questionnaires and the response rate was 71.4%.

In this survey, the proportion of male employees was 46.5% and female employees was 53.5%. In terms of age distribution, 17.1% of the employees were 18–25 years old, 33.9% were 26–35 years old, 42.6% were 36–45 years old, 6.3% were 45–50 years old, and 1.2% were 51 years old and above. In terms of education level, 43.1% had high college degrees, 49.3% had bachelor’s degrees, and 7.6% had master’s degrees or above. Around 40.5% of employees had less than 5 years of working experience, 12.4% had 5–10 years, 20.2% had 11–15 years, 16.3% had 16–20 years, and 10.7% had more than 20 years.

### Measures

Since all the mature scales published in top journals were used in this paper, double-blind and standard translation method were adopted to ensure the consistency of the questionnaire. In addition, well-known domestic management scholars were invited to test and revise all the Chinese scales. Then, some graduate students sent the questionnaire to the employees for a small sample test. The results showed that the reliability and validity of the questionnaire were high and can be further distributed to large samples. A Likert 5-point scale was used to measure the following variables, ranging from “1 = strongly disagree” to “5 = strongly agree.”

#### Electronic communication during nonwork time

This scale was measured with Ma et al. (2003) ’s 3 items scale. A Likert 5-point scale was used, ranging from “1 = never” to “5 = very often.” For example, “How often do I communicate with others for work matters through the APPs (e.g., WeChat, Tencent Meeting) during non-working hours?.” The Cronbach’s α for this scale was 0.824.

#### Employee withdrawal behavior

Lehman and Simpson’s scale (1992) was used to assess employee withdrawal behavior. This scale includes 12 items such as “I chat with colleagues about nonwork-related topics.” Acceptable Cronbach’s α for this scale was 0.741.

#### Job engagement

The job engagement scale from Lanaj et al. (2014) with 3 items was used for job engagement analysis. Sample items of this scale such as “I am full of energy in my work.” The Cronbach’s α for this scale was 0.862.

#### Regulatory emotional self-efficacy

Caprara et al.’s scale (2008) was used to assess regulatory emotional self-efficacy. This scale includes 12 items such as “I was able to keep my chin up in the face of sharp criticism.” The Cronbach’s α for this scale was 0.927.

#### Controlling variables

Referring to previous studies on electronic communication during nonwork time and employee withdrawal behavior (Blau, 1985; Zimmerman et al., 2016; Becker et al., 2018), gender, age, education level and working years of employees were selected as controlling variables.

### Analytical strategy

This study used SPSS 23.0 and AMOS 23.0 Software to assess the (inverted) U-shaped model. The data analysis process included three steps. First, descriptive analysis was conducted, including mean, standard deviation (SD) and correlation coefficient. Second, confirmatory factor analysis (CFA) was conducted to verify the validity and reliability of the measurement model. Third, a hierarchical regression analysis was used to test the hypothesized model.

## Result analysis

### Descriptive analysis

The mean, SD and correlation coefficient of each variable are shown in Table 1. The electronic communication during nonwork time was negatively correlated with employee withdrawal behavior (*r* = −0.087, *p* < 0.05), and positively correlated with job engagement (*r* = 0.292, *p* < 0.01). In addition, job engagement was negatively corelated with employee withdrawal behavior (*r* = −0.341, *p* < 0.01). The correlation analysis results provided initial evidence for the hypotheses.

**Table 1:** Descriptive statistic and correlation analysis for each variable.

Variable	Mean	SD	1	2	3	4
1. Electronic communication during nonwork time	2.55	0.89	1			
2. Job engagement	1.48	0.53	0.292**	1		
3. Employee withdrawal behavior	4.26	0.76	−0.087*	−0.341**	1	
4. Regulatory emotional self-efficacy	4.08	0.84	0.005	−0.077	0.395**	1

* *p* < 0.05;

** *p* < 0.01.

### Measurement model

We conducted a CFA to validate the measurement model. As showed in Table 2, the fit indices of proposed model (four-factor model: electronic communication during nonwork time, regulatory emotional self-efficacy, job engagement, and employee withdrawal behavior) was better than other alterative models ( $\chi^{2}$ = 1495.27, df = 521, $\chi^{2}/df$ = 2.87, CFI = 0.90, GFI = 0.93, TLI = 0.90, RMSEA = 0.07). The results showed that the four-factor model can be used for subsequent hypothesis testing.

**Table 2:** The results of confirmatory factor analysis.

Model	Factors	$\chi^{2}$	*df*	$\chi^{2}/df$	CFI	GFI	TLI	RMSEA
One-factor model	ECNT + JE + RESE + EWB	5480.72	527	10.40	0.45	0.54	0.41	0.14
Two-factor model 1	ECNT + JE + RESE, EWB	4764.71	526	9.06	053	0.61	0.50	0.13
Two-factor model 2	ECNT, JE + RESE + EWB	4827.59	526	9.18	0.52	0.56	0.49	0.13
Three-factor model 1	ECNT, JE + RESE, EWB	3007.76	524	5.74	0.61	0.64	0.58	0.11
Three-factor model 2	ECNT + JE, RESE, EWB	2363.24	524	4.51	0.76	0.79	0.75	0.09
Three-factor model 3	ECNT, JE, RESE + EWB	2075.04	524	3.96	0.83	0.85	0.83	0.08
Three-factor model 4	ECNT + RESE, JE, EWB	1828.76	524	3.49	0.87	0.88	0.87	0.08
Four-factor model	ECNT, JE, RESE, EWB	1495.27	521	2.87	0.90	0.93	0.90	0.07

ECNT, electronic communication during nonwork time; JE, job engagement; RESE, regulatory emotional self-efficacy; EWB, employee withdrawal behavior.

### Tests of hypotheses

This study used the three conditions of “U-shaped relationship test” proposed by Haans et al. (2016) to explore the U-shaped relationship between electronic communication during nonwork time and employee withdrawal behavior. They mentioned that the positive (inverted) U-shaped relationship exits, when: Condition 1: the square term of independent variable is positively (negatively) related to dependent variable; Condition 2: when the value of the independent variable is minimum, the slope of the curve is negative (positive), and when the value of the independent variable is maximum, the slope is positive (negative). Condition 3: the inflection point of the curve should be in the range of the independent variable.

**Figure 2:**
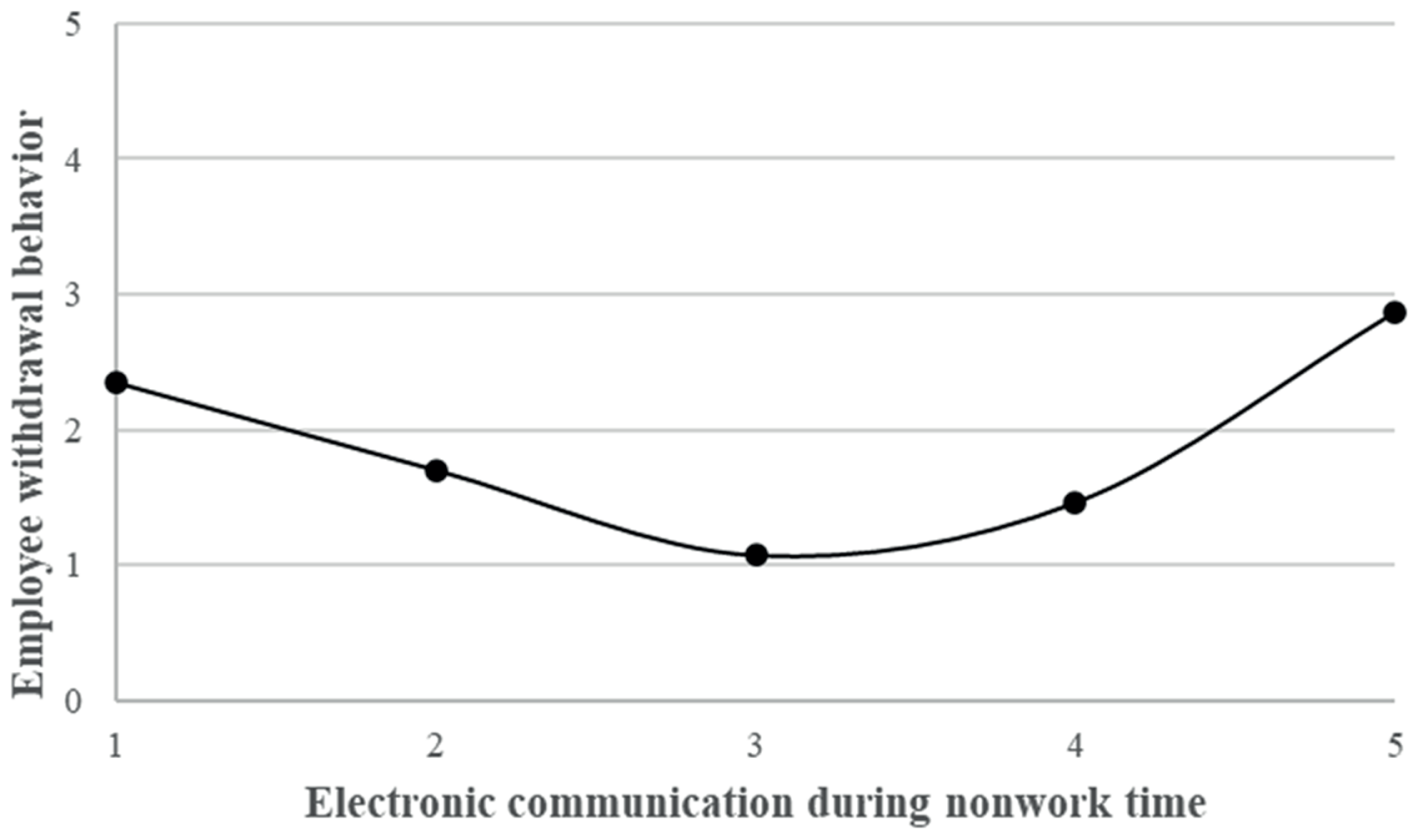
The U-shape relationship between ECNT and employee withdrawal behavior.

Assume that the regression equation of electronic communication during nonwork time to job engagement is: $y=\beta_{0}+\beta_{1}x+\beta_{2}x^{2}$ . SPSS 23.0 software was used and the results are shown in Table 3. In Model 1, the square term of electronic communication during nonwork time had a significant positive influence on employee withdrawal behavior after controlling employee’s gender, age, education level and working years ( $\beta_{2}$ = 0.51, *p* < 0.01), and Condition 1 was met. Then, the value of the slope $k=\beta_{1}+2\beta_{2}x$ was calculated. According to Model 1, $\beta_{1}$ = −0.58， $\beta_{2}\;$ = 0.51, and the range of normalized independent variable was [−1.75, 2.76]. Therefore, when the value of electronic communication during nonwork time was minimum ( $x_{\min}$ = −1.75), then k < 0; when the value of independent variable was maximum ( $x_{\max}$ = 2.76), then k > 0. Condition 2 was met. Finally, we calculated the inflection point ( $x=-\beta_{1}/2\beta_{2})$ of the curve and found that its value was 0.57, which belongs to the interval [−1.75, 2.76], and Condition 3 was met. Then, Hypothesis 1 was supported. It indicated that there was a U-shaped relationship between electronic communication during nonwork time and employee withdrawal behavior. That is, less electronic communication during nonwork time can reduce employee withdrawal behavior. In contrast, as the frequency of electronic communication during nonwork time increases, it leads to more employee withdrawal behavior (as shown in Figure 2).

**Table 3:** The results of hierarchical regression analysis.

Variables	Model 1	Model 2	Model 3	Model 4
	Employee withdrawal behavior	Job engagement	Employee withdrawal behavior	Job engagement
Gender	0.001	−0.09	−0.03	−0.08
Age	0.22	−0.14	0.17*	−0.14
Education level	−0.06	0.12	−0.02	0.12
Working years	−0.14	0.21**	−0.07	0.21**
ECNT	−0.58**	0.79***	−0.33	0.67***
ECNT^2^	0.51**	−0.55**	0.35*	−0.37*
Job engagement			−0.33***	
RESE				−0.07
ECNT × RESE				0.35
ECNT^2^ × RESE				−0.38*
*R* ^2^	0.049	0.148	0.141	0.157
*F*	4.39***	14.76***	11.89***	10.47***

* *p* < 0.05;

** *p* < 0.01;

*** *p* < 0.001.

**Figure 3:**
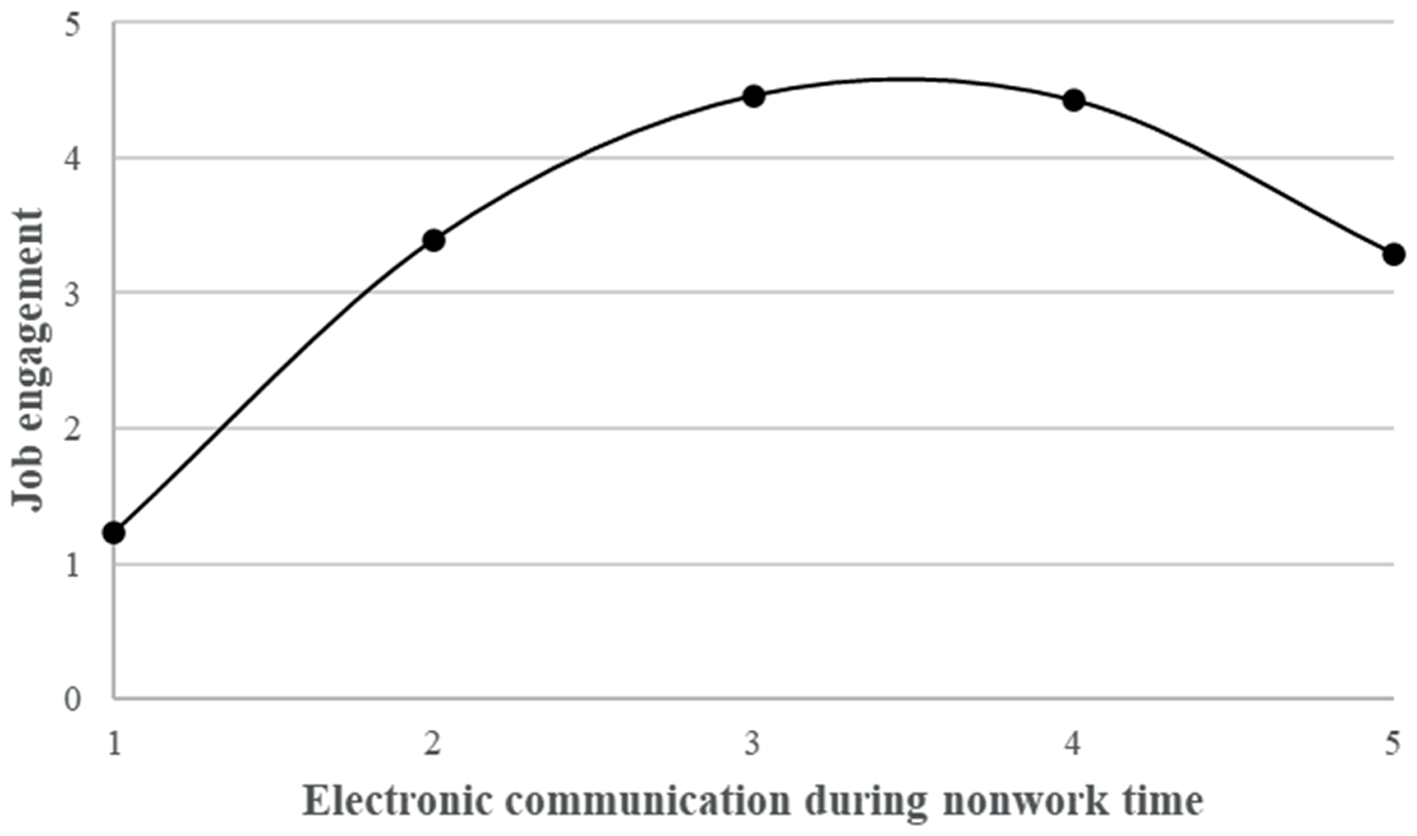
The inverted U-shape relationship between ECNT and job engagement.

Similarly, the inverted U-shaped relationship between electronic communication during nonwork time and job engagement ( $m=\beta_{3}+\beta_{4}x+\beta_{5}x^{2}$ ) was also analyzed according to the above inverted U-shaped test conditions. The results of Model 2 in Table 3 showed that, after controlling employee’s gender, age, education level and working years, the square term of electronic communication during nonwork time had a significant negative correlation with job engagement ( $\beta_{5}$ = −0.55, *p* < 0.01). When electronic communication during nonwork time was minimum ( $x_{\min}$ = −1.75), the value of slope k > 0; and when the value of independent variable was maximum ( $x_{\max}$ = 2.76), then k < 0. The inflection point of the curve was 0.72, which fell in the interval [−1.75, 2.76]. Condition (1) to (3) were met. It indicated that there was an inverted U-shaped relationship between electronic communication during nonwork time and job engagement (as showed in Figure 3). And Hypothesis 2 was supported.

In model 3, there was a significant negative relationship between job engagement and employee withdrawal behavior (*β* = −0.33, *p* < 0.001), while the relationship between the square term of independent variable (ECNT2) and employee withdrawal behavior decreased significantly, with a coefficient of *β* = 0.35. According to Baron and Kenny’s (1986) mediating variable test, job engagement has a partial mediating effect between electronic communication during nonwork time and employee withdrawal behavior. However, Hayes (2017) proposed that Baron’s mediation test method may have bias when U-shaped relationship exist between variables. Therefore, the Bootstrapping method proposed by Hayes (2017) was further used to test the mediating effect of job engagement between electronic communication during nonwork time and employee withdrawal behavior. The results are shown in Table 4. Job engagement had a significant mediating effect (*β* = −0.23), and the 95% confidence interval (−0.36, −0.13) did not include zero. Thus, the mediating effect of job engagement was statistically significant. Then, Hypothesis 3 was supported.

**Table 4:** The results of mediation analysis.

Effect test	*β*	SD	Sig.	95% confidence interval	
				Lower	Upper
Total effect	−0.28	0.15	<0.05	−0.58	−0.01
Direct effect	−0.05	0.03	<0.05	0.001	0.11
Mediating effect	−0.23	0.06	<0.001	−0.36	−0.13

Finally, this research constructed the equation $m=\beta_{6}+\beta_{7}x+\beta_{8}x^{2}+\beta_{9}xw+\beta_{10}x^{2}w+\beta_{11}w$ to test the moderating effect of regulatory emotional self-efficacy. Haans proposed that the moderating effect in the inverted U-shaped relationship has two different effects: one is that the curve moves to the left/right at the inflection point, and the other is that the curve slope becomes steeper/gentler. According to Model 4 in Table 3, $\beta_{7}$ = 0.67, $\beta_{8}$ = −0.37, $\beta_{9}$ = 0.35, $\beta_{10}$ = −0.38. The value of inflection point $x^{*}$ = $\beta_{7}\beta_{10}-\beta_{8}\beta_{9}$ = −0.13 < 0, indicating the curve of high regulatory emotional self-efficacy shifted from right to the left. Then, $\beta_{10}$ = −0.38 (*p* < 0.05) indicated that the moderating variable makes the inverted U-shaped curve steeper. And Hypothesis 4 was supported.

**Figure 4:**
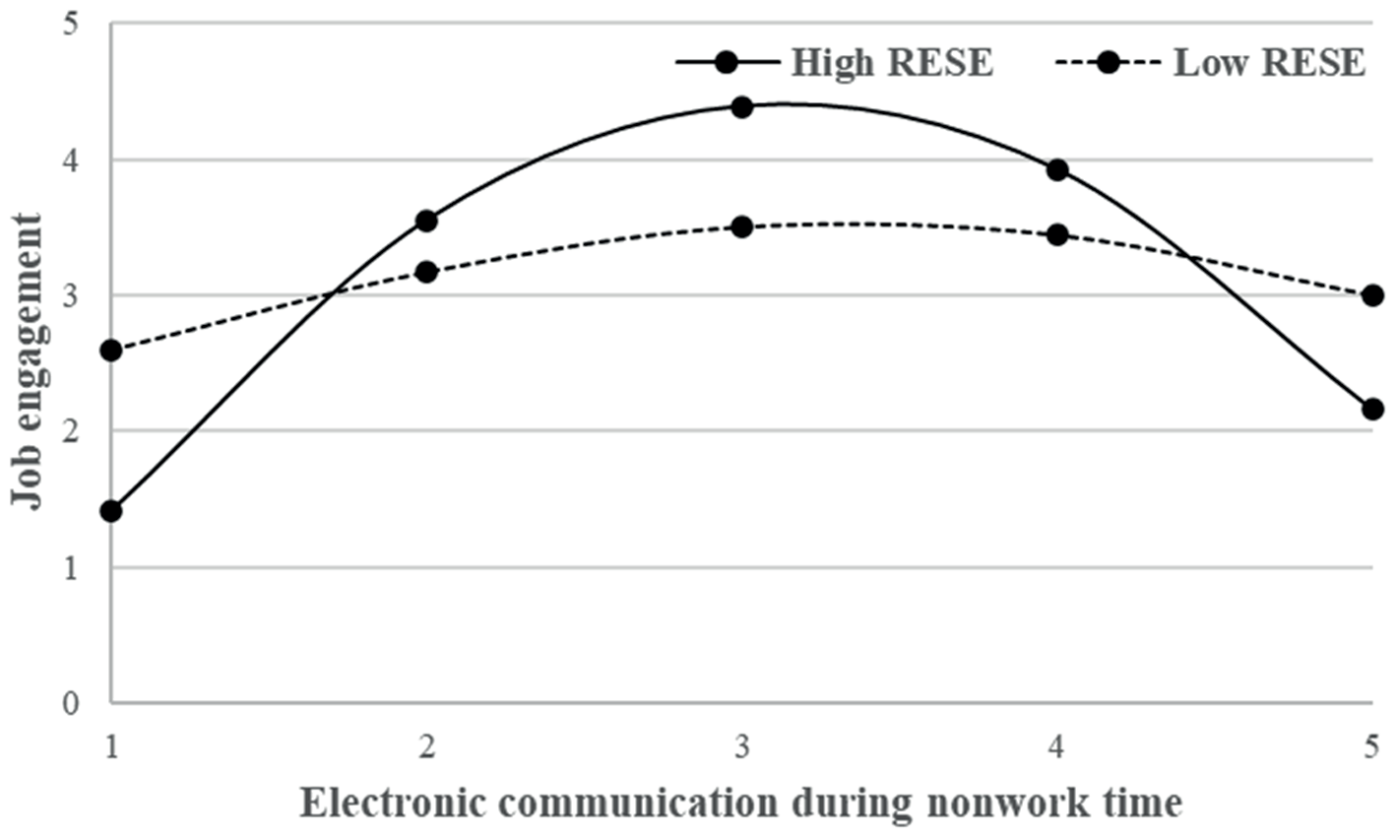
The moderating effect of regulatory emotional self-efficacy.

Moreover, a Simple slope test proposed by Aiken and West (1991) was used to analyze the moderating effect of regulatory emotional self-efficacy. As showed in Figure 4, the different level of modeator (Mean ± 1SD) has different effect on relationship between electronic communication during nonwork time and job engagement. When the frequency of electronic communication during nonwork time was low, employees with high emotional regulatory self-efficacy had a stronger positive effect on the relationship between electronic communication during nonwork time and job engagement than those with low regulatory emotional self-efficacy. When the frequency of electronic communication during nonwork time was too high, the negative effect was stronger for employees with high regulatory emotional self-efficacy.

## Discussion

This study investigated the relationship between electronic communication during nonwork time and employee withdrawal behavior based on the JD-R theory. The mediating role of job engagement and the moderating role of regulatory emotional self-efficacy was also tested. This study draws the following three conclusions.

First, although a large number of literatures have explored the relationship between electronic communication during nonwork time and individual perception and behavior based on the Western cultural context (Butts et al., 2015; Becker et al., 2018; Reinke and Ohly, 2021), we still have some limitations on this topic under the cultural background of collectivism, “Guanxi” in China. This study builds upon previous work by the scholars (Derks et al., 2016; Ferguson et al., 2016; Xie et al., 2018), by looking at the effect of electronic communication during nonwork time on employee withdrawal behavior in the Chinese cultural context. The results indicate that the relationship between electronic communication during nonwork time on employee withdrawal behavior is more complicated, reflecting the nature of Chinese employees’ desire to build relationships and access resources through electronic communication during nonwork time. Specifically, moderate level of electronic communication during nonwork time is more likely to reduce employee withdrawal behavior. In China, individuals put more emphasis on collectivism, dedication and “Guanxi,” and they would devote more time and energy to work. This efficient pattern of working, electronic communication during nonwork time increases the frequency of communication between employees and work-related parties, strengthens the connection between employees and other people, impels employees to establish a sound social network (Ren and Chadee, 2017). All these are helpful to enhance employees’ positive emotions at work, improve their organization identification (Lv et al., 2022), so as to alleviate their psychological withdrawal and reduce employee withdrawal behavior (Knippenberg et al., 2007). However, when the level of electronic communication during nonwork time increased from moderate to high, employee withdrawal behavior was found to increase. Information overload, extra workload, and time and energy spent in non-working time make employees fall into job burnout, resulting in individual anxiety, lassitude and other negative mental states (Xie et al., 2018; Von-Bergen and Bressler, 2019). Therefore, the negative impact of electronic communication during nonwork time was significantly increased in this study.

Second, we also found that electronic communication during nonwork time has an inverted U-shaped effect on job engagement. This means that employees with moderate level of electronic communication during nonwork time are more likely to engage in organizations than those with low level of electronic communication during nonwork time. This is line up with the scholars’ research (Syrek et al., 2018). A possible explanation for this might be that electronic commination during nonwork time helps employees obtain a sense of control over work progress, increases their job engagement and makes them more willing to focus on work and produce high performance (Syrek et al., 2018). In contrast, when electronic commination during nonwork time rose from moderate to high, we found that employees’ job engagement decreases accordingly. That means that employees need to spend a lot of personal time to deal with job tasks. This extra workload lengthens the working hours and increases the intensity of the work, which leads to the continuous consumption of employees’ positive emotions and behaviors. As a result, they will gradually lose their enthusiasm and reduce the motivation in the future work (Bandura et al., 2003; Fonner and Roloff, 2012), which will lead to the decrease of job engagement.

Third, this study suggests that regulatory emotional self-efficacy moderate the inverted U-shaped relationship between electronic communication during nonwork time and job engagement. This finding consistent with the previous studies which highlight the importance of regulatory emotional self-efficacy in strengthening the impact of job characteristics on employees’ cognitions (Alessandri et al., 2015; Yuan et al., 2018). The result show that the inverted U-shaped effect is stronger when the employee has high regulatory emotional self-efficacy. Especially, the positive moderating effect is more pronounced with moderate level of electronic communication during nonwork time. Individuals with higher regulatory emotional self-efficacy can ease emotional tension and maintain self-regulation mechanism to a certain extent (Zhang et al., 2019). They can effectively perceive and actively make use of the job resources. These help individuals better work to realize their self-worth, thus enhancing employees’ job engagement.

### Theoretical implication

The followings are the theoretical contributions of this study. First, it enriches the research on the electronic communication during nonwork time and employee withdrawal behavior in the Chinese cultural context. Previous studies have shown that electronic communication during nonwork time has a positive or negative impact on individual behavior. However, most studies focused on work family conflict, emotion exhaustion based on Western culture (Butts et al., 2015; Becker et al., 2018; Reinke and Ohly, 2021), with a few on employee withdrawal behavior based on Chinese traditional cultural values. This study explored the relationship between electronic communication during nonwork time and employee withdrawal behavior in Chinese cultural context, such as collectivism, dedication, and “Guanxi,” greatly enriching the relevant topic in the above fields.

Secondly, this study answers the reason for the inconsistent conclusions of previous studies on the relationship between electronic communication during nonwork time and individual behavior based on the JD-R theory. Most of the previous studies were based on Work Family Boundary theory, Ego Depletion theory, Conservation of Resources (Becker et al., 2018; Von-Bergen and Bressler, 2019; Zhang et al., 2019), etc., and examined the negative or positive effects of electronic communication during nonwork time, respectively. However, this study reveals that electronic communication during nonwork time can be regarded as both job resources and job demands. Therefore, its influence on individual behavior may be nonlinear. Empirical evidence showed that electronic communication during nonwork time had a U-shaped effect on employee withdrawal behavior through the dual path of job resources and job demands. These findings not only expand the empirical research on the double-edged effect of electronic communication during nonwork time on employee withdrawal behavior, but also enrich the application of JD-R theory.

Third, the mediating effect of job engagement and the moderating effect of regulatory emotional self-efficacy were investigated, further enriching the research on the influence mechanism of electronic communication during nonwork time on employee withdrawal behavior. Although the previous literature has confirmed that there was a significant relationship between electronic communication during nonwork time and job engagement, only the positive or negative relationship between them has been discussed (Fujimoto et al., 2016; Syrek et al., 2018). This study reveals that the relationship between electronic communication during nonwork time and job engagement is more complicated, which leads to the complexity of the relationship between electronic communication during nonwork time and employee withdrawal behavior. Moreover, the inverted U-shaped effect of electronic communication during nonwork time on job engagement is stronger when the regulatory emotional self-efficacy of employee is higher. These differences are particularly evident at moderate levels of electronic communication during nonwork time. Our findings not only provide a boundary condition for the double-edged effect of electronic communication during nonwork time on job engagement. At the same time, it also expands the theoretical application of the regulatory emotional self-efficacy.

### Practical implications

Firstly, for managers, it is necessary to pay close attention to the negative effects of electronic communication during nonwork time. It should be realized that this type of work-related communication may objectively cause the loss of job resources, reduce the job engagement of employees, and then cause the lack of vitality and motivation, and finally lead to the employee withdrawal behavior. Comprehensively understanding and effectively using electronic communication during nonwork time have become an issue that managers need to take seriously. These require managers to master the “way” and “frequency” of electronic communication during nonwork time, and avoid the negative impact as much as possible. When the frequency of electronic communication during nonwork time of employees is high, managers need to care for employees timely. Besides, the organization can also pay employees for the task completed in non-working times to avoid withdrawal behavior caused by the perception of unfairness.

Secondly, as employees, they need to constantly adapt to the changes in work situation brought about by the development of science and technology. When electronic communication during nonwork time becomes inevitable, employees should be proactive in adapting to the increasingly blurred boundaries between work and family. For example, employee should arrange their time effectively to reduce the negative impact of electronic communication during nonwork time. At the same time, employees should also recognize the positive effect of electronic communication during nonwork time, which can provide them with job resources and help improve their job engagement, and thus improve the quality of work.

Thirdly, individuals with higher regulatory emotional self-efficacy are able to ease emotional tension and maintain self-regulation mechanism. They can effectively deal with the job resources and job demands provided by electronic communication during nonwork time. Therefore, in organizational human resource management practices, it is necessary to pay more attention to employees’ regulatory emotional self-efficacy. For example, in the process of recruitment and selection, the HR managers should not only examine candidates’ skills and knowledge, but assess their ability to regulate emotions. Modern evaluation technique, interview and other methods could be used to select employees with high regulatory emotional self-efficacy, so as to further improve employees’ job engagement and reduce withdrawal behavior.

### Limitations and future directions

Although the double-edged mechanism between electronic communication during nonwork time and employee withdrawal behavior was confirmed by the U-shaped model, this study also has several limitations. First, this study focused on electronic communication during nonwork time and employee withdrawal behavior in the Chinese cultural context, a more emphasis on collectivism, dedication, “Guanxi” culture than Western culture. Future research should examine the relationship between electronic communication during nonwork time and employee withdrawal behavior in other cultures. Secondly, the self-report data of employees were used in the data analysis process, which may lead to Common Method Bias (Podsakoff et al., 2003). In the future, longitudinal or multi-source data should be used to examine the relationship. Thirdly, this study investigated the mediating effect of job engagement. Other potential mediating variables such as organizational identification, power distance, and organizational commitment should also be considered in the future research (Lee et al., 2000; Edwards, 2005; Al-Jabari and Ghazzawi, 2019). Fourth, this study investigated the moderating effect of regulatory emotional self-efficacy as a whole. It can be known from the literature that this variable contains three sub-dimensions (Caprara et al., 2008). Future studies could analyze the different moderating effects of regulatory emotional self-efficacy from these three sub-dimensions.

## Conclusion

This study investigates the double-edged impact of electronic communication during nonwork time on employee withdrawal behavior in the Chinese cultural context, and also confirms the mediating effect of job engagement and moderating effect of regulatory emotional self-efficacy. These findings provide new insights on the electronic communication during nonwork time in organizations to reduce employee withdrawal behavior.

## Data availability statement

The raw data supporting the conclusions of this article will be made available by the authors, without undue reservation.

## Author contributions

GL: conceptualization, investigation, writing original draft, and funding acquisition. ML: data analysis and methodology. JY: writing review and editing. QW: data collection and formal analysis. All authors contributed to the article and approved the submitted version.

## Funding

This research was funded by the Project of National Natural Science Foundation of China Project (71801017 and 72002016), the Project of Beijing Social Science (18GLC064), and Beijing Knowledge Management Institute (5212210983).

## Conflict of interest

The authors declare that the research was conducted in the absence of any commercial or financial relationships that could be construed as a potential conflict of interest.

## Publisher’s note

All claims expressed in this article are solely those of the authors and do not necessarily represent those of their affiliated organizations, or those of the publisher, the editors and the reviewers. Any product that may be evaluated in this article, or claim that may be made by its manufacturer, is not guaranteed or endorsed by the publisher.
